# Elastocaloric-effect-induced adiabatic magnetization in paramagnetic salts due to the mutual interactions

**DOI:** 10.1038/s41598-021-88778-4

**Published:** 2021-05-03

**Authors:** Lucas Squillante, Isys F. Mello, Antonio C. Seridonio, Mariano de Souza

**Affiliations:** 1 IGCE - Physics Department, São Paulo State University (Unesp), Rio Claro, SP 13506-900 Brazil; 2 Department of Physics and Chemistry, São Paulo State University (Unesp), Ilha Solteira, SP 15385-000 Brazil

**Keywords:** Materials science, Physics

## Abstract

The temperature change under adiabatic stress, i.e., the elastocaloric effect, is a well-understood phenomenon and of particular interest due to its potential application in alternative ways for refrigeration. Here, we demonstrate that in the regime of low-temperatures (a few mK) real paramagnets can be magnetized when compressed adiabatically without applied magnetic field. Such adiabatic magnetization is a genuine many-body problem, stemming from the inherent dipolar mutual interactions between adjacent magnetic moments. We showcase experimental setups to carry out adiabatic magnetization and thus to access such a subtle effect. Perspectives of further investigations by controlling the mutual interactions in Bose–Einstein condensates in magnetic insulators and dipolar spin-ice systems via the adiabatic increase of temperature are also presented. Yet, we discuss the connection between the elastic Grüneisen parameter and the shift on the critical temperature of second-order phase transitions under adiabatic stress, as well as its connection with the Ehrenfest relation.

## Introduction

The understanding of the behavior of magnetic excitations in solids continues to be a topic of broad interest. Indeed, the interplay between magnetism and superconductivity^1,2^, a proper description and the search for materials aiming to maximize the magnetocaloric effect^3,4^, magnetic-field-induced quantum phase transitions^5–7^, and exotic excitations like magnetic skyrmions^8,9^ are currently fertile fields of research. It has been almost a century since paramagnetic salts have been employed for cooling in the adiabatic demagnetization process^10^. It is well-known that the intrinsic mutual interactions between adjacent magnetic moments are the limiting factor in the attainment of lower temperatures, typically a few mK, using this process^7,10^. Essentially, such mutual interactions generate an effective local magnetic field $B_{loc}$ that prevents further demagnetization for magnetic fields lower than $B_{loc}$ and, as a consequence, the system cannot be cooled below such a temperature. The many-body effects emerging from the mutual interactions show up when both the thermal and the magnetic dipolar energy^10,11^ become comparable. Recently, we have reported on the influence of such many-body effects in preventing the realization of a genuine zero-field quantum phase transition in real paramagnets^7^. Also, we have reported on the possibility of performing adiabatic magnetization^7^ by only manipulating the mutual interactions between spins. At this point, it is worth mentioning that the adiabatic magnetization here discussed is achieved without an external magnetic field and thus it differs from the known adiabatic magnetization usually reported in the literature concerning the so-called inverse magnetocaloric effect^12,13^. Essentially, the idea consists in increasing the temperature adiabatically^10,14^, so that the system is magnetized in order to maintain the entropy constant^7^. This is particularly true based on the fact that in an adiabatic process the total entropy should be held constant, while the entropy associated with the various excitations involved can be either decreased or increased^15^. Also, considering the reversibility of an adiabatic process, the adiabatic magnetization can be restarted as long as the system lies in a temperature range, in which the magnetic excitations associated with the mutual interactions are relevant. Here, we discuss the basic concepts of Thermodynamics associated with the adiabatic temperature increase, as well as experimental setups for the realization of elastocaloric-effect-induced adiabatic magnetization in paramagnetic salts. Yet, we discuss the possibility of exploring many-body effects in other magnetic systems via the adiabatic increase of temperature.

## Temperature change due to an adiabatic compression

Based on concepts of Thermodynamics, the temperature of a system can be increased, without heat exchange with the surroundings, via adiabatic compression^10,16^. In such a case, upon applying pressure the volume *v* varies quasi-statically. Next, we derive the expression for temperature change due to an adiabatic pressurization. We start recalling the first law of Thermodynamics^16,17^:

1 $$\begin{aligned} dQ = dU + dW, \end{aligned}$$

where d*Q* is the infinitesimal heat variation, d*U* the infinitesimal internal energy variation, and *dW* the infinitesimal work performed on the system upon applying external pressure. In a quasi-static process, the internal energy variation d*U* can be written as^17^:

2 $$\begin{aligned} d U = \left( \frac{\partial U}{\partial T}\right) _{v}d T, \end{aligned}$$

where *T* is the temperature. The term $\left( \frac{\partial U}{\partial T}\right) _{v}$ in Eq. (2) can be recognized as the heat capacity at constant volume $c_v$^17^ and thus:

3 $$\begin{aligned} d U = c_vd T. \end{aligned}$$

The infinitesimal work *dW* performed on the system is given by $dW = -pd v$^10,16,17^, where *p* is the applied pressure. Employing the relation for an adiabatic process $p = -\left( \frac{\partial U}{\partial v}\right) _S$^17^, where *S* is the entropy, the infinitesimal work *dW* can be written as follows:

4 $$\begin{aligned} dW = -pd v = \left( \frac{\partial U}{\partial v}\right) _{S}d v = \left( \frac{\partial U}{\partial S}\right) _{v}\left( \frac{\partial S}{\partial v}\right) _Td v. \end{aligned}$$

The term $\left( \frac{\partial U}{\partial S}\right) _v = T$ in Eq. (4) is the canonical definition of temperature^10^. Also, employing the Maxwell-relation $\left( \frac{\partial S}{\partial v}\right) _T = \left( \frac{\partial p}{\partial T}\right) _v$^17^, Eq. (4) becomes:

5 $$\begin{aligned} dW = T\left( \frac{\partial p}{\partial T}\right) _{v}d v. \end{aligned}$$

Replacing Eqs. (3) and (5) into Eq. (1):

6 $$\begin{aligned} d Q = c_{v}d T+T\left( \frac{\partial p}{\partial T}\right) _{v}d v. \end{aligned}$$

Recalling that d*Q* = *T*d*S*^10,16,17^ we write^17,18^:

7 $$\begin{aligned} Td S = c_vd T + T\left( \frac{\partial p}{\partial T}\right) _vd v, \end{aligned}$$

Since d$Q = T$d*S* an adiabatic compression at a fixed *T* means that d$Q = 0$ and thus implying that *S* must remain constant (d$S = 0$) during this process. Therefore, Eq. (7) becomes:

8 $$\begin{aligned} c_vd T = - T\left( \frac{\partial p}{\partial T}\right) _vd v. \end{aligned}$$

Employing the relation^17^:

9 $$\begin{aligned} \left( \frac{\partial p}{\partial T}\right) _v\left( \frac{\partial T}{\partial v}\right) _p\left( \frac{\partial v}{\partial p}\right) _T = -1 \Rightarrow \left( \frac{\partial p}{\partial T}\right) _v = -\frac{1}{\left( \frac{\partial T}{\partial v}\right) _p\left( \frac{\partial v}{\partial p}\right) _T} \end{aligned}$$

and using the Thermodynamic relations for the isothermal compressibility $\kappa _T = - \frac{1}{v}\left( \frac{\partial v}{\partial p}\right) _T$^17^ and the volumetric thermal expansion $\beta = \frac{1}{v}\left( \frac{\partial v}{\partial T}\right) _p$^17^, Eq. (9) becomes:

10 $$\begin{aligned} \left( \frac{\partial p}{\partial T}\right) _v = \frac{\beta }{\kappa _T}. \end{aligned}$$

Thus, replacing Eq. (10) into (8) we have:

$$\begin{aligned} c_vd T = - \frac{T\beta }{\kappa _T}d v. \end{aligned}$$

Resulting thus in the key expression:

11 $$\begin{aligned} d T_S = -\frac{T\beta }{c_v\kappa _T}d v, \end{aligned}$$

where d$T_S$ refers to the infinitesimal temperature variation in an adiabatic compression. Equation 11 indicates that upon adiabatically compressing a solid, its volume is reduced (d$v < 0$) and thus the temperature is increased adiabatically (d$T_S > 0$) starting in a certain *T*. The temperature increase due to an adiabatic volume change here discussed is isentropic and thus a reversible adiabatic process. The very same mathematical analysis as in Eq. (11) can be performed upon analysing the increase of temperature due to an adiabatic increase of pressure. Now, we make use of the following thermodynamic relation^14^:

12 $$\begin{aligned} d S(T,p) = \left( \frac{\partial S}{\partial T}\right) _p d T + \left( \frac{\partial S}{\partial p}\right) _T d p. \end{aligned}$$

The derivative on the first term of the right side of Eq. (12) is $(\partial S/\partial T)_p = c_p/T$, where $c_p$ is the heat capacity at constant pressure. Also, employing the Maxwell-relation $(\partial S/\partial p)_T = - (\partial v/\partial T)_p$^17^ Eq. (12) becomes:

13 $$\begin{aligned} d S = \frac{c_p}{T}d T - \left( \frac{\partial v}{\partial T}\right) _pd p. \end{aligned}$$

Considering a reversible adiabatic process [$d S(T,p) = 0$], Eq. (13) reads:

14 $$\begin{aligned} \frac{c_p}{T}d T_S = \left( \frac{\partial v}{\partial T}\right) _pd p_S, \end{aligned}$$

where d$p_S$ is the adiabatic pressure change. Thus, we achieve:

15 $$\begin{aligned} d T_S = \frac{\beta v T}{c_p}d p_S. \end{aligned}$$

Essentially, Eq. (15) is similar to Eq. (11). Both Eqs. (11) and (15) can be employed to either describe an adiabatic compression (d$v <0$, d$p >0$) or expansion (d$v > 0$, d$p < 0$). However, in this work we have focused on the case of an adiabatic increase of temperature (d$T_S > 0$), i.e., an adiabatic compression. Note that in Eq. (15) the adiabatic temperature increase is written in terms of an infinitesimal adiabatic pressure change d$p_S$. Thus, in the light of Eq. (15) the temperature is increased due to an adiabatic application of pressure. At this point, we recall that the cooling of a system using pressure as the tuning parameter is the so-called barocaloric effect. The physical parameter that quantifies the barocaloric effect is the Grüneisen parameter $\Gamma _p = 1/T(\partial T/\partial p)_S$^19^. Interestingly enough, by manipulating Eq. (15) we obtain:

16 $$\begin{aligned} \left( \frac{\partial T}{\partial p}\right) _S = \frac{\beta v T}{c_p}. \end{aligned}$$

Multiplying both sides of Eq. (16) by 1/*T*, gives us:

17 $$\begin{aligned} \frac{\beta v }{c_p} = \frac{1}{T}\left( \frac{\partial T}{\partial p}\right) _S = \Gamma _p. \end{aligned}$$

Note that Eq. (17) embodies naturally the definition of $\Gamma _p$^19^. Experimentally, one of the ways of increasing the temperature adiabatically upon pressurization is by employing an adiabatic application of stress, which is discussed in the next Section.

## Uniaxial stress application

**Figure 1:**
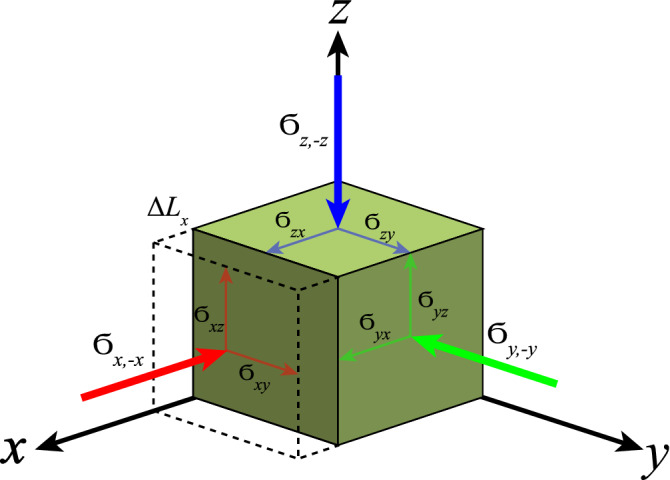
Uniaxial stress application in a solid. Schematic representation of the 9 stress components: 3 normal stresses ($\sigma _{x,-x}$, $\sigma _{y,-y}$, and $\sigma _{z,-z}$) and 6 shear stresses ($\sigma _{xy}$, $\sigma _{xz}$, $\sigma _{yx}$, $\sigma _{yz}$, $\sigma _{zx}$, and $\sigma _{zy}$). All the stress components can be represented in the Voigt’s abbreviated notation $\sigma _{ij}$^20^, as discussed in more details in the main text. The dashed lines indicate the length variation $\Delta L_x$ when an uniaxial compressive stress $\sigma _{x,-x}$ is applied. Figure based on Ref.^21^. The application of uniaxial stress can be used for the adiabatic magnetization of a paramagnetic salt, cf. depicted in Fig. 2 and discussed in the main text.

Another way of promoting an adiabatic increase of temperature lies in the application of an uniaxial stress under adiabatic conditions^18^. The entropy variation in an adiabatic process with respect to the temperature and applied stress $\sigma$ is given by^18^:

18 $$\begin{aligned} d S = \left( \frac{\partial S}{\partial T}\right) _\sigma d T + \left( \frac{\partial S}{\partial \sigma _{ij}}\right) _{T,\sigma }d \sigma _{ij} = 0, \end{aligned}$$

where $\sigma _{ij}$ represents the Voigt’s abbreviated notation^20^ for the stress components, the index *i* corresponds to the plane normal on which the stress is applied and *j* the direction of the applied stress^21^. Experimentally, the application of uniaxial stress on the specimen may be accompanied by an undesired strain gradient due to the bending of the sample^22^. Thus, in order to avoid such a strain gradient, a symmetric mounting of the stress application setup is required, as discussed in Ref.^22^. Essentially, the symmetric mounting lies in attaching a rigid cap foil on top of the sample’s edges, to ensure that both lower and upper surfaces of the sample’s edges are firmly secured and thus a homogeneous stress application is attainable^18,22^, cf. Fig. 1. In our analysis, we consider that all stress components are kept constant except for one of the normal stresses since we are interested in the case of an uniaxial applied stress, cf. Fig. 1. Thus, considering that $(\partial S/\partial \sigma _{ij})_{T,\sigma } = \alpha _{ij}$, where $\alpha _{ij}$ is the thermal expansion coefficient in the Voigt’s abbreviation, and $(\partial S/\partial T)_{\sigma } = c_{\sigma }/T$, where $c_{\sigma }$ is the heat capacity at constant stress. Thus, rewriting Eq. (18) we achieve:

19 $$\begin{aligned} d T_S = -\frac{T}{c_{\sigma }}\alpha _{ij}d \sigma _{ij}. \end{aligned}$$

Since we focus on the case of an adiabatic compression, d$\sigma _{ij}$ is recognized as the compressive stress, which is conventionally negative^21^. Thus, d$T_S$ in Eq. (19) is positive meaning an adiabatic increase in temperature under an adiabatic uniaxial compressible stress. Thus, Eq. (19) can be rewritten as follows:

20 $$\begin{aligned} \frac{1}{T}\left( \frac{\partial T}{\partial \sigma _{ij}}\right) _S = \frac{\alpha _{ij}}{c_{\sigma }} = \Gamma _{ec}. \end{aligned}$$

Following discussions in our previous work^19^, we define $\Gamma _{ec}$ as the elastic Grüneisen parameter, which quantifies the temperature change due to an adiabatic uniaxial strain application. A key aspect regarding Eq. (19) refers to the experimental difficulties posed in achieving adiabatic conditions. In this regard, the oscillating strain technique can be employed, cf. depicted in Fig. 3. Using this technique, the strain frequency should be tuned in a time-scale lower than the thermal relaxation time of the system^18^. Thus, the system is strained more quickly than it exchanges heat with its surroundings and thus an adiabatic strain on the specimen can be attained. At this point, we consider that the entropy variation d*S* also depends on the magnetization change d*M* and thus we write:

21 $$\begin{aligned} d S(T,M,\sigma ) = \left( \frac{\partial S}{\partial T}\right) _{M,\sigma }d T + \left( \frac{\partial S}{\partial M}\right) _{T,\sigma }d M + \left( \frac{\partial S}{\partial \sigma _{ij}}\right) _{T,M}d \sigma _{ij}. \end{aligned}$$

Because of a reversible adiabatic process $d S(T,M,\sigma ) = 0$ and thus:

22 $$\begin{aligned} -\left( \frac{\partial S}{\partial M}\right) _{T,\sigma }d M = \left( \frac{\partial S}{\partial T}\right) _{M,\sigma }d T + \left( \frac{\partial S}{\partial \sigma _{ij}}\right) _{T,M}d \sigma _{ij}. \end{aligned}$$

Employing the Maxwell relation $\left( \frac{\partial S}{\partial M}\right) _T = -\left( \frac{\partial B}{\partial T}\right) _S$^17^, Eq. (22) becomes:

23 $$\begin{aligned} \left( \frac{\partial B}{\partial T}\right) _Sd M = \left( \frac{\partial S}{\partial T}\right) _{M,\sigma _{ij}}d T + \left( \frac{\partial S}{\partial \sigma }\right) _{T,M}d \sigma _{ij}. \end{aligned}$$

Equation 23 embodies the principle of the adiabatic magnetization under the application of uniaxial stress. In order to hold the entropy constant when the temperature is increased adiabatically, there has to be a variation of the magnetization d*M* to compensate the entropy change associated with both d$T_S$ and d$\sigma _{ij}$, as well as a change of the magnetic field *B* in respect to the adiabatic temperature variation. It is clear that *B* should be recognized as $B_{loc}$, since there is no external magnetic field acting on the system. Essentially, an adiabatic application of $\sigma _{ij}$ in a paramagnetic system implies in an adiabatic temperature increase and to hold *S* constant, the system is spontaneously magnetized adiabatically by d*M*. Note that we have considered that a paramagnetic insulator presents a finite magnetization in the absence of an external magnetic field when $T \rightarrow$ 0 K due to the intrinsic presence of $B_{loc}$, being thus its ground-state a ferromagnetic phase, cf. discussions presented in Ref.^7^. Therefore, the infinitesimal magnetization d*M* in Eq. (23) is due to the magnetic energy increase associated with the mutual interactions in order to keep the entropy constant as a response to an adiabatic increase of temperature. Based on such discussions, it is tempting to infer the concept of positive and negative temperatures^10,23,24^ in terms of adiabatic deformations. However, the requirements for such are not fulfilled^24^.

## The Grüneisen parameter and strain-dependent second-order phase transitions

As discussed by us in Ref.^19^, the Grüneisen parameter is enhanced close to any critical point due to the intrinsic entropy accumulation near it and, as previously discussed, the elastic Grüneisen parameter quantifies the elastocaloric effect. As discussed in the next, the elastic Grüneisen parameter incorporates the shift in the critical temperature $T_c$ of a second-order phase transition due to the application of stress. Under adiabatic conditions, such a shift in $T_c$ occurs because the temperature variation of the specimen due to the elastocaloric effect has to compensate the entropy change associated with the strain^18^. We make use of the expression for the entropy variation of an infinitesimal strain $d \varepsilon$ under adiabatic conditions (d$S = 0$) close to a second-order phase transition^18^:

24 $$\begin{aligned} d S = -\frac{c_{\sigma }^{(cr)}}{T}\left( \frac{d T_c}{d \varepsilon }\right) _Td \varepsilon + \frac{c_{\sigma }}{T}d T = 0, \end{aligned}$$

where $c_{\sigma }^{(cr)}$ is the critical contribution to the heat capacity at constant stress. The first term on the right side of Eq. (24) associates the entropy change due to $(d T_c/d \varepsilon )_T$ and the second term accounts for the entropy variation due to the elastocaloric effect of the specimen^18^. Rearranging Eq. (24), we have^18^:

25 $$\begin{aligned} \left( \frac{d T}{d \varepsilon }\right) _S = \frac{c_{\sigma }^{(cr)}}{c_{\sigma }}\left( \frac{d T_c}{d \varepsilon }\right) _T. \end{aligned}$$

Equation 25 relates the elastocaloric effect to the corresponding shift in $T_c$^18^. Note that $\Gamma _{ec}$ can be determined in terms of $d \varepsilon$ upon multiplying both sides of Eq. (25) by 1/*T*. Since Eq. (25) accounts for the critical contribution to the heat capacity near $T_c$, it is associated with the critical contribution to the elastic Grüneisen parameter $\Gamma ^{cr}_{ec}$, namely:

26 $$\begin{aligned} \Gamma ^{cr}_{ec} = \frac{1}{T}\left( \frac{d T}{d \varepsilon }\right) _S = \frac{1}{T}\frac{c_{\sigma }^{(cr)}}{c_{\sigma }}\left( \frac{d T_c}{d \varepsilon }\right) _T. \end{aligned}$$

Interestingly, $\Gamma ^{cr}_{ec}$ quantifies the shift in $T_c$ in the vicinity of a second-order phase transition due to an adiabatic strain. If $(\mathrm {d}T/\mathrm {d}\varepsilon )_S > 0$ in Eq. (26) implies that the temperature of the system is adiabatically increased in response to the strain, which is the case of an adiabatic compressible stress ($\sigma _{ij} < 0$), cf. Eq. (19). Thus, $(\mathrm {d}T_c/\mathrm {d}\varepsilon )_T$ is also positive, i.e., $T_c$ is shifted to higher temperatures in response to the adiabatic strain. Based on similar arguments, if $(\mathrm {d}T/\mathrm {d}\varepsilon )_S < 0$ in Eq. (26), $T_c$ is shifted to lower temperatures. It is well-known that $c_{\sigma }^{(cr)}$ is enhanced close to $T_c$ and thus, based on Eq. (26), $\Gamma _{ec}^{cr}$ is enhanced as well. Hence, a pronounced elastocaloric effect is expected close to a second-order phase transition. This is in perfect agreement with our recent work about giant caloric effects close to critical points, cf. discussed in Ref.^19^. Next, we discuss the connection between the Grüneisen parameter in the vicinity of $T_c$ and the celebrated Ehrenfest relation^25^. Note that close to $T_c$, Eq. (19) can be easily rewritten in terms of the thermal expansion and heat capacity jumps, $\Delta \alpha _{ij}$ and $\Delta c_{\sigma }$, which correspond to the difference between critical and ordinary contributions to the thermal expansion coefficient and heat capacity, respectively. Hence, we have:

27 $$\begin{aligned} \mathrm {d}T_c = -T_c\frac{\Delta \alpha _{ij}}{{\Delta {c_{\sigma }}}} \mathrm {d}\sigma _{ij}. \end{aligned}$$

Rearranging Eq. (27), we achieve^26,27^:

28 $$\begin{aligned} \left( \frac{\mathrm {d} T_c}{\mathrm {d}\sigma _{ij}}\right) _S=-T_c\frac{\Delta \alpha _{ij}}{\Delta {c_{\sigma }}}, \end{aligned}$$

which has some resemblance with the Ehrenfest relation^25^. Equation 28 indicates the $T_c$ variation under adiabatic stress. More specifically, Eq. (28) takes into account the critical contribution to the elastic Grüneisen parameter $\Gamma _{ec}^{cr}$, which reads:

29 $$\begin{aligned} \Gamma _{ec}^{cr}=\frac{1}{T_c}\left( \frac{\mathrm {d} T_c}{\mathrm {d} \sigma _{ij}}\right) _S = -\frac{\Delta \alpha _{ij}}{\Delta {c_{\sigma }}}. \end{aligned}$$

In this case, $\Gamma _{ec}^{cr}$ quantifies the shift in $T_c$ under the application of an adiabatic stress analogously to the shift in $T_c$ under application of pressure in the well-known Ehrenfest relation. Although Eq. (29) has already been reported in Refs.^26,27^, its connection with the Grüneisen parameter is still lacking. It is worth mentioning that such an analysis can be extended to other cases, such as the magneto-caloric and the electro-caloric effects, namely^6,7,19^:

30 $$\begin{aligned} \Gamma _{mag}^{cr}=\frac{1}{T_c}\left( \frac{\mathrm {d} T_c}{\mathrm {d} B}\right) _S = -\frac{\Delta \alpha _{B}}{\Delta {c_{B}}}, \end{aligned}$$

and

31 $$\begin{aligned} \Gamma _{ece}^{cr}=\frac{1}{T_c}\left( \frac{\mathrm {d} T_c}{\mathrm {d} E}\right) _S = -\frac{\Delta \alpha _{E}}{\Delta {c_{E}}}, \end{aligned}$$

where $\Gamma _{mag}^{cr}$ and $\Gamma _{ece}^{cr}$ are the critical contributions to the magneto-caloric and electro-caloric Grüneisen parameters, and *E* is the electric field. Thus, in an analogy with the canonical Ehrenfest relation, Eqs. (29), (30), and (31) quantify the $T_c$ shift of a second-order phase transition under the adiabatic application of stress, magnetic field, or electric field, respectively.

## Adiabatic magnetization by only manipulating the mutual interactions between spins

The magnetic interactions between adjacent magnetic moments in a real paramagnet lead to a finite effective local magnetic field $B_{loc}$, which is usually about 0.01 T considering an average distance between magnetic moments of 5Å^7^. By cooling the paramagnetic system down to temperatures, in the range in which the energy associated with the magnetic interactions are relevant (usually $T < 6$ mK)^7^, many-body effects set in. In such a temperature range it is possible to increase the temperature adiabatically, cf. previous discussions. Essentially, in order to keep the entropy constant there has to be an increase of the local field to compensate such temperature increase^7^, i.e., the system is magnetized. For the sake of completeness, we recall some relevant results reported by us in Ref.^7^. The microscopic treatment of the mutual interactions requires a many-body approach, which can be described by the Hamiltonian^7,28,29^:

32 $$\begin{aligned} H=-J'\sum _{(ij)}\vec {S}_i^{z_i}\cdot \vec {S}_j^{z_j}+Dr_{nn}^3 \sum _{i>j} \frac{\vec {S}_i^{z_i}\cdot \vec {S}_j^{z_j}}{|\vec {r}_{ij}|^3}-\frac{3(\vec {S_i}^{z_i}\cdot \vec {r}_{ij})(\vec {S_j}^{z_j}\cdot \vec {r}_{ij})}{|\vec {r}_{ij}|^5}, \end{aligned}$$

where $J'$ is the magnetic coupling constant, $\vec {S}$ is the spin vector oriented along the local $z_i$ Ising $<111>$ axis, *i* and *j* refer to the two sites of the lattice, $\vec {r}$ is the position vector, $D = (\mu _0\mu ^2)/(4\pi r_{nn}^3)$, $\mu _0$ is the vacuum permeability, $\mu$ the magnetic moment, and $r_{nn}$ is the distance between nearest-neighbor spins. The second term of the Hamiltonian (Eq. 32) embodies the magnetic energy associated with the interaction between a single magnetic moment and its nearest neighbors. The Hamiltonian of Eq. (32) is key in understanding the adiabatic magnetization here discussed, since it takes into account the microscopic character of the mutual interactions responsible to give rise to $B_{loc}$. It turns out that when the temperature is increased adiabatically, the mutual interactions are altered in order to increase the magnetic energy and to keep the entropy constant. In our analysis, we employ a simple mean-field-type (molecular-field) approach to treat the mutual interactions, which is discussed in the next. At this point, the understanding of the adiabatic magnetization requires a treatment in terms of the uncertainty principle. In order to keep the entropy constant during the adiabatic magnetization process, the magnetic energy $U_{mag}$ of the paramagnetic system has to be changed, which is given by^30^:

33 $$\begin{aligned} U_{mag} = -\frac{m_J}{\sqrt{J(J+1)}}\mu B_{loc} = -\mu B_{loc}\cos {\phi }, \end{aligned}$$

where $m_J$ is the is the magnetic quantum number, *J* the total angular momentum quantum number, and $\phi$ is the angle between $\mu$ and $B_{loc}$. Employing $U_{mag}$, the energy uncertainty $\Delta E$ can be calculated by the expression^10^:

34 $$\begin{aligned} \Delta E = U_{mag} - E, \end{aligned}$$

where *E* is the average magnetic energy. Plugging Eq. (33) into Eq. (34) and $E = \mu _B B_{loc}N\tanh {\left( \frac{\mu _B B_{loc}}{k_B T}\right) }$^10^, where *N* is the number of particles, we have^7^:

35 $$\begin{aligned} \Delta E = \mu _BB_{loc}\left[ N\tanh {\left( \frac{\mu _BB_{loc}}{k_B T}\right) }-\cos {\phi }\right] . \end{aligned}$$

**Figure 2:**
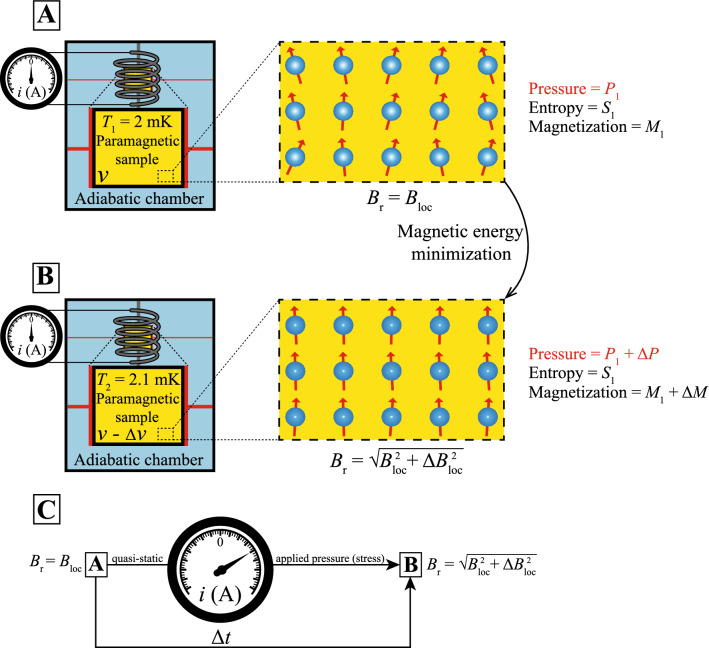
Schematic representation of the steps for performing the adiabatic magnetization of a paramagnetic specimen^7^. (**A**) A sample with volume *v* is inserted inside a coil, which is attached to an ampere meter, into an adiabatic chamber and the temperature is decreased to $T_1 = 2$ mK, being the sample under pressure $p_1$, with entropy $S_1$, and magnetization $M_1$. External magnetic fields are absent and thus the resulting magnetic field $B_r = B_{loc}$ due to the intrinsic mutual interactions between adjacent magnetic moments (represented by the red arrows). The dashed lines represent a zoom of the paramagnetic sample inside the coil and the spins inside the sample. (**B**) A quasi-static pressure, more specifically the stress as depicted in Fig. 3, is applied so that the volume is reduced by a factor $\Delta v$, leading thus to an adiabatic increase of the sample temperature to $T_2 = 2.1$ mK. The sample is now under a pressure ($p_1 + \Delta p$) and it is magnetized by a factor $\Delta M$ in order to hold the entropy $S_1$ constant. Since the magnetization of the sample is increased (minimization of the energy, cf. discussions in the main text), the spins are slightly vertically aligned since now the resulting field is $B_r \simeq \sqrt{{B_{loc}}^2 + {\Delta B_{loc}}^2}$. (**C**) During the time interval $\Delta t$ from process A to B (quasi-static applied pressure, i.e., stress cf. Fig. 3), the magnetic field is increased from $B_{loc}$ to $\sqrt{{B_{loc}}^2 + {\Delta B_{loc}}^2}$ and thus the magnetic flux is changed over $\Delta t$ inducing an electromotive force, which in turn is associated with a finite electrical current, which can be measured by the ampere meter. Some parts of this figure were created employing templates available in Ref.^31^.

In Eq. (35), $\Delta E$ is minimized when $\cos {\phi } \rightarrow 1$^7^. Since the adiabatic magnetization is performed during a quasi-static process, this means that the uncertainty in time $\Delta t$ should be maximized, while $\Delta E$ is minimized. The condition for $\Delta E$ to be minimized lies in varying the total angular momentum projection^7^. This is attainable by increasing only the *z*-axis projection of the total angular momentum vector $\vec {J}$. The consequence of the enhancement of the $\vec {J}$ projection in the *z*-axis is that the magnetic energy is decreased and thus the system is magnetized^7^, meaning that the angle $\phi$ between $\mu$ and $B_{loc}$ is lowered. In summary, the genesis of the increment $\Delta B_{loc}$ in the adiabatic magnetization process is given by the increase of the $\vec {J}$ projection in the *z*-direction. This is one of the key results reported in Ref.^7^. In general terms, in the adiabatic magnetization process (Fig. 2) of the mutual interactions, the temperature is adiabatically increased from $T_1$ to $T_2$ and thus we can write^7^:

36 $$\begin{aligned} T_2 = T_1\frac{\sqrt{B_{loc}^2 + {\Delta B_{loc}}^2}}{B_{loc}}, \end{aligned}$$

where $\mu _B$ is the Bohr magneton, $k_B$ is the Boltzmann constant, and $\Delta B_{loc}$ is the magnetic field increment that will emerge into the system to compensate the adiabatic increase of temperature. The term $\Delta B_{loc}$ refers to the adiabatic magnetization by only manipulating the mutual interactions of the system upon adiabatically increasing its temperature. Then, $\Delta B_{loc}$ can be determined by the simple expression^7^:

37 $$\begin{aligned} \Delta B_{loc} = B_{loc}\sqrt{\frac{T_2^2}{T_1^2} - 1}. \end{aligned}$$

## Experimental realization of the adiabatic magnetization

**Figure 3:**
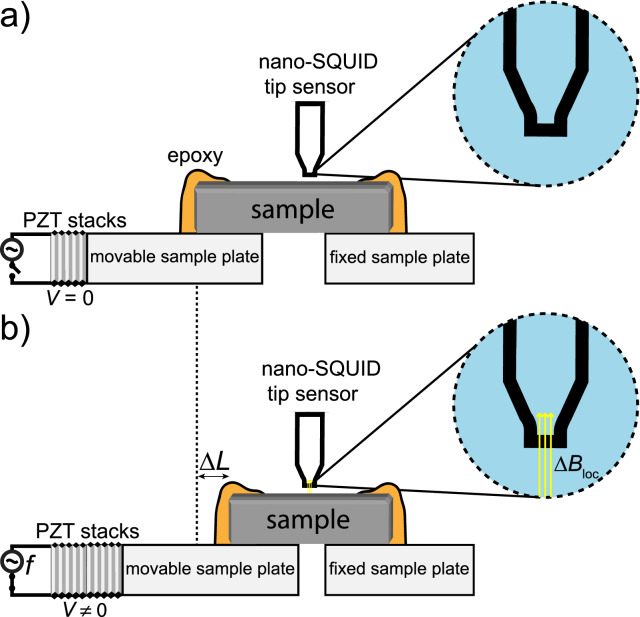
Schematic representation of the adiabatic magnetization process in a paramagnetic salt employing an experimental setup for the application of uniaxial stress. The sample is attached to both movable and fixed plates by using epoxy. A nano-SQUID tip sensor is placed close to the sample. Lead zirconate titanate (PZT) piezoelectric stacks are attached to the movable plate. In (**a**) there is no applied A.C. voltage *V* and thus no deformation of the PZT stacks takes place. In (**b**) an A.C. voltage $V \ne 0$ is applied to the PZT stacks at a given frequency *f* making the PZT stacks to deform. Hence, the sample is uniaxially compressed leading to a variation of its length *L* by $\Delta L$. The adiabatic regime can be achieved upon tuning *f*. The employed frequency should be higher than the inverse relaxation time $\tau ^{-1}$ of the system, since $\tau$ represents the time scale of the heat exchange between the sample and its surroundings, cf. Ref.^18^. In this process, the temperature of the system is adiabatically increased and thus an adiabatic magnetization of the sample takes place, which is detected by the attached nano-SQUID tip sensor. Figure prepared based on Refs.^18,22,37^. More details in the main text.

In order to carry out the adiabatic magnetization of a paramagnetic system, it must be isolated from the surrounding in a low-temperature condition ($T < 6$ mK) and pressure, which experimentally can be the stress, cf. illustrated in Fig. 3, must be quasi-statically applied to increase its temperature adiabatically. Upon increasing the stress as shown in Fig. 3, in such a quasi-static process, the temperature will be increased adiabatically and then the paramagnetic system will be magnetized by a term $\Delta B_{loc}$, as previously discussed, in order to keep the entropy constant^7^.

## SQUID

Another possible way of measuring $\Delta B_{loc}$ would be employing a nano-SQUID on a tip^32^, which presents outstanding sensitivity to measure the magnetic field generated by a single magnetic moment. In the present case, we are interested in measuring the local field during the adiabatic magnetization process. Essentially, the presence of a magnetic flux $\Phi$ through the SQUID affects its corresponding superconducting electrical current. Such magnetic flux depends on the magnetic field acting on the SQUID and the surface area $S'$ of the superconducting loop. The magnetic flux $\Phi$ through the SQUID is given by^33^:

38 $$\begin{aligned} \Phi = \int _{S'}\vec {B}\cdot d \vec {S'} = n\left( \frac{h}{2e}\right) , \end{aligned}$$

where *n* is an integer number, *h* is Planck’s constant, and *e* the fundamental electron charge. The factor (*h*/2*e*) is the quantum of magnetic flux $\Phi _0$ and it dictates the SQUID’s sensitivity. Considering the conditions of the adiabatic temperature increase from $T_1$ to $T_2$ (cf. Fig. 2) for the realization of the adiabatic magnetization, we estimate $\Delta B_{loc} \approx$ 3.2 mT^7^. In the particular case of a nano-SQUID on a tip^32^, given the fact that $\Phi$ depends on $S'$, the sensitivity for detecting magnetic fields is compromised by the nanometer size of such a device. However, the spatial resolution of the device is improved and its sensitivity to magnetic dipoles increased, i.e., the nano-SQUID on a tip is extremely sensitive to small magnetic moments^32^, enabling thus the detection of $\Delta B_{loc}$ generated from the adiabatic magnetization process in a paramagnetic system. Given the high-sensitivity of the SQUID’s critical current to both temperature and magnetic field, the nano-SQUID on a tip is also a very accurate scanning cryogenic thermal sensor^34–36^. Thus, employing such a device, a very sensitive thermal imaging of energy dissipation of nanoscale processes can be carried out^34–36^. Hence, the thermal mapping of the adiabatic heating during the adiabatic magnetization can be carried out employing the oscillating strain experimental setup proposed in Ref.^18^ with an attached nano-SQUID on tip^32^, cf. depicted in Fig. 3. The relevant parameters involving the use of a nano-SQUID on a tip for measuring the adiabatic magnetization here discussed, such as the width of the measured signal for a particular frequency of the PZT stacks A.C. voltage, shall depend on the particular physical properties of the investigated system.

## Adiabatic increase of temperature in interacting systems

The adiabatic increase of temperature upon applying pressure (stress, as depicted in Fig. 3) can lead to other investigations in the frame of many-body systems. There is a subtle difference between applying pressure/stress (Fig. 3) to a system and then sweeping its temperature non-adiabatically with the case of increasing its temperature adiabatically upon applying external pressure/stress (Fig. 3). When adiabatic pressurization takes place in an interacting system, it will rearrange itself as a whole due to the many-body effects in order to hold the entropy constant, which obviously is not the case for non-adiabatic conditions. Thus, employing adiabatic temperature changes, the many-body character of various interacting systems can be explored in an unprecedented way. This is particular true for Bose–Einstein condensates (BEC) in magnetic insulators^38^ and dipolar spin-ice^29^ systems, just to mention a few examples. In the specific case of the BEC in a magnetic insulator reported in Ref.^38^, the adiabatic increase of temperature in such a system will change the energy $\varepsilon$ of a triplon (Eq. 2 of Ref.^38^) and, as a consequence, the density of triplons will be altered due to the so-called Zeeman term $-g\mu _B BS^{z}$ in the triplon energy, where *g* is the gyromagnetic ratio, $S^{z}$ is the spin projection in the *z* direction, and *z* is the direction of the external magnetic field. Considering that the resulting magnetic field of the system depends on both the external applied magnetic field and $B_{loc}$ inherent to this particular system, the adiabatic increase of temperature will change $B_{loc}$, which in turn will change the density of triplons as well. Note that in this particular case, both an external magnetic field and $B_{loc}$ are considered^7^, which is not the case for the adiabatic magnetization due to the mutual interactions discussed previously. Hence, the adiabatic increase of temperature can be seen as an alternative way for investigating the system’s interactions and its phase diagram. This is a simple example of an investigation of the many-body character in interacting systems that can be carried out by employing an adiabatic increase of temperature. Yet, we point out that a similar case of the adiabatic magnetization for the Brillouin-like paramagnet, performed in the temperature range below which is associated with the relevance of the magnetic dipolar mutual interactions, can be carried out for the case of electric dipoles. More specifically, when considering the temperature regime where the interactions between electric dipoles are relevant, the Langevin equation^39^ can be employed following the approach reported in Ref.^7^ to demonstrate that when the temperature is adiabatically increased, a spontaneous adiabatic electric polarization of the system takes place, analogously to the adiabatic magnetization.

## Conclusions

We have revisited the basic concepts associated with the increase of temperature under adiabatic application of hydrostatic pressure and uniaxial stress. Experimentally, the attainable mechanism to increase the temperature adiabatically is the application of stress. Following our previous work^7^, we have proposed experimental setups for detecting the magnetic response under adiabatic conditions. Furthermore, we have proposed an alternative way for exploring various exotic phases of matter, like BEC in magnetic insulators and spin-ice, upon changing the temperature adiabatically. Yet, we have discussed that the elastic Grüneisen parameter quantifies the shift in the critical temperature $T_c$ of a second-order phase transition under an adiabatic strain, as well as its connection with the Ehrenfest relation.

## Methods

All the figures presented in this work were created employing the software Adobe Illustrator Version CC 2017 and a few templates available in Ref.^31^ were used in the creation of the figures.

## References

[1] Mathur ND (1998). Magnetically mediated superconductivity in heavy fermion compounds. Nature.

[2] Bert JA (2011). Direct imaging of the coexistence of ferromagnetism and superconductivity at the LaAlO$$_3$$/SrTiO$$_3$$ interface. Nat. Phys..

[3] Pecharsky VK, Gschneidner KA (1999). Magnetocaloric effect and magnetic refrigeration. J. Magn. Magn. Mater..

[4] Tishin AM, Spichkin YI (2003). The Magnetocaloric Effect and its Applications.

[5] Sachdev S (2011). Quantum Phase Transitions.

[6] Gomes GO, Squillante L, Seridonio AC, Ney A, Lagos RE, de Souza M (2019). Magnetic Grüneisen parameter for model systems. Phys. Rev. B.

[7] Squillante L, Mello IF, Gomes GO, Seridonio AC, Lagos-Monaco RE, Stanley HE, de Souza M (2020). Unveiling the physics of the mutual interactions in paramagnets. Sci. Rep..

[8] Fert A, Reyren N, Cros V (2017). Magnetic skyrmions: advances in physics and potential applications. Nat. Rev. Mater..

[9] Peng L (2020). Controlled transformation of skyrmions and antiskyrmions in a non-centrosymmetric magnet. Nat. Nanotechnol..

[10] Baierlein R (1999). Thermal Physics.

[11] Blundell S (2001). Magnetism in Condensed Matter.

[12] Krenke T (2005). Inverse magnetocaloric effect in ferromagnetic Ni–Mn–Sn alloys. Nature.

[13] Moya X (2007). Cooling and heating by adiabatic magnetization in the Ni$$_{50}$$Mn$$_{34}$$In$$_{16}$$ magnetic shape-memory alloy. Phys. Rev. B.

[14] Sonntag SE, Borgnakke C, Wylen GJV (2003). Fundamentals of Thermodynamics.

[15] Landau LD, Lifshitz EM (1959). Statistical Physics.

[16] Callen HB (1960). Thermodynamics.

[17] Stanley HE (1971). Introduction to Phase Transitions and Critical Phenomena.

[18] Ikeda MS (2019). AC elastocaloric effect as a probe for thermodynamic signatures of continuous phase transitions. Rev. Sci. Instrum..

[19] Squillante, L., Mello, I. F., Seridonio, A. C. & de Souza, M. Giant caloric effects close to any critical end point. Preprint at arXiv:2003.13060 (2020).

[20] Barron THK, Collins JG, White GK (1980). Thermal expansion of solids at low temperatures. Adv. Phys..

[21] Meyers MA, Chawla KK (2009). Mechanical Behavior of Materials.

[22] Hicks CW, Barber ME, Edkins SD, Brodsky DO, Mackenzie AP (2014). Piezoelectric-based apparatus for strain tuning. Rev. Sci. Instrum..

[23] Purcell EM, Pound RV (1951). A nuclear spin system at negative temperature. Phys. Rev..

[24] Ramsey NF (1956). Thermodynamics and statistical mechanics at negative absolute temperatures. Phys. Rev..

[25] Ehrenfest P (1933). Phasenumwandlungen im üblichen und erweiterten Sinn, classifiziert nach den entsprechenden Singularitäten des thermodynamischen Potentiales. Proc. Acad. Sci. Amst..

[26] Mori K, Hayashi M (1972). Effect of a two-dimensional pressure on the Curie points of triglycine sulphate and Rochelle salt. J. Phys. Soc. Jpn..

[27] Moin PB (2016). Ehrenfest equations for second-order phase transition under hydrostatic and uniaxial stresses. Ph. Transit..

[28] Feynman RP (1998). Statistical Mechanics.

[29] Bramwell ST, Gingras M (2001). J.-P. Spin ice state in frustrated magnetic pyrochlore materials. Science.

[30] Guimarães AP (1998). Magnetism and Magnetic Resonance in Solids.

[31] Vectorized images available at https://www.vecteezy.com.

[32] Vasyukov D (2013). A scanning superconducting quantum interference device with single electron spin sensitivity. Nat. Nanotechnol..

[33] Ashcroft NW, Mermin ND (1976). Solid State Physics.

[34] Halbertal D (2016). Nanoscale thermal imaging of dissipation in quantum systems. Nature.

[35] Halbertal D (2017). Imaging resonant dissipation from individual atomic defects in graphene. Science.

[36] Marguerite A (2019). Imaging work and dissipation in the quantum Hall state in graphene. Nature.

[37] Gannon L (2015). A device for the application of uniaxial strain to single crystal samples for use in synchrotron radiation experiments. Rev. Sci. Instrum..

[38] Giamarchi T, Rüegg C, Tchernyshyov O (2008). Bose–Einstein condensation in magnetic insulators. Nat. Phys..

[39] Kittel C (2005). Introduction to Solid State Physics.

